# CBX2 Inhibits Neurite Development by Regulating Neuron-Specific Genes Expression

**DOI:** 10.3389/fnmol.2018.00046

**Published:** 2018-02-28

**Authors:** Xi Gu, Xuemin Wang, Dazhuang Su, Xiaohong Su, Lifang Lin, Shuji Li, Qiaoqi Wu, Shuhu Liu, Peidong Zhang, Xinhong Zhu, Xiaodan Jiang

**Affiliations:** ^1^Department of Neurosurgery, Zhujiang Hospital, Southern Medical University, The National Key Clinical Specialty, The Engineering Technology Research Center of Education Ministry of China, Guangdong Provincial Key Laboratory on Brain Function Repair and Regeneration, Guangzhou, China; ^2^Department of Neurobiology, Southern Medical University, Guangzhou, China; ^3^School of Traditional Chinese Medicine, Southern Medical University, Guangzhou, China

**Keywords:** CBX2, miR-124, neuronal gene expression and regulation, neuronal development, GAP-43

## Abstract

Polycomb group (PcG) proteins regulate the epigenetic status of transcription regulatory states during development. Progression from pluripotency to differentiation requires the sequential activation and repression of different PcG target genes, however, the relationship between early patterning signals, PcG expression, and the development of the central nervous system is still unclear. Using various models of neuronal differentiation, we provide evidence that CBX2 is a negative regulator of neuronal differentiation. Knock-down of CBX2 expression promotes neurite development, while overexpression of CBX2 inhibits neurite development. Further, we found that CBX2 is a direct target gene of miR-124. During neuronal differentiation, CBX2 was decreased while miR-124 was increased. Mechanistically, CBX2 directly interacts with the promoter region of several neuro-associated genes and regulates their expression. We found that the neuron-specific GAP-43 gene could contribute to the stimulating effect on neurite development associated with inhibition of CBX2.

## Introduction

Cell differentiation is mainly achieved via repression of the important genes required for cell pluripotency, with simultaneously triggering of a cascade of epigenetic changes and de-repressing of the expression of lineage-specific genes (Camahort and Cowan, 2012). First discovered in *Drosophila* (Jacobs and van Lohuizen, 1999), the Polycomb group (PcG) proteins were recognized as important regulators of differentiation and do so by forming and maintaining repressive chromatin states (Di Croce and Helin, 2013). The PcGs mainly form two protein complexes, the Polycomb repressive complex 2 (PRC2) and the Polycomb repressive complex 1 (PRC1). The PRC2 complex generally contains the Ezh1/2, Suz12, and Eed. In the classical model, through its catalytic subunit EZH2, PRC2 trimethylates histone H3 at lysine 27 (H3K27me3), thereby inhibiting the expression of its target genes (Cao et al., 2002). Then H3K27me3 can then be recognized by the N-terminal chromodomain of one of the five CBX proteins (CBX2, 4, 6, 7, 8), which are members of PRC1 (Kaustov et al., 2011). Once bound to H3K27me3, the CBX proteins can recruit other components of PRC1 to chromatin via protein–protein interactions. Recruitment of PRC1 can further stimulate transcriptional inhibition through various mechanisms such as histone H2A ubiquitination (H2AK119Ub) and chromatin compaction (Francis et al., 2004; Eskeland et al., 2010; Gao et al., 2012).

Recently, the roles played by the PcGs proteins in the development of central nervous system (CNS) have started to emerge. A study showed that the PcG proteins epigenetically suppress the bHLH proneural gene Neurogenin1 (Ngn1) locus during the astrogenic phase, thus triggering the neurogenic-to-astrogenic fate switching of neural precursor cells and regulate the duration of the neurogenic phase during early development of CNS (Hirabayashi et al., 2009). The neuron-restrictive silencer factor/RE1-silencing transcription factor (NRSF/REST), a zinc finger repressor that function as a negative regulator of neuronal genes in non-neuronal cells and in neural stem cells and progenitors prior to neuronal differentiation (Chong et al., 1995; Schoenherr and Anderson, 1995) was found to recruit the PRC1 complex by interacting with the CBX family proteins, with CBX2 shows the strongest interaction (Ren and Kerppola, 2011). CBX2, a component of the mammalian Polycomb repressive complex 1 (PRC1), have been show to involved in proper body patterning (Lau et al., 2017), growth and sex determination of the gonad (Biason-Lauber et al., 2009). Despite these advances, how the PcGs are regulated in the process of neural development remains unclear.

MicroRNAs, a class of small non-coding RNAs that regulate gene expression at the post-transcription level, are now known to play a key role in neuronal development and plasticity (Olde Loohuis et al., 2012). miR-124, a nervous system-specific microRNA, has been confirmed to promote neuronal differentiation by directly regulating multiple genes. Expression of miR-124 is gradually increased during development of the CNS (Deo et al., 2006; Krichevsky et al., 2006). Inhibiting the activity of miR-124 in neurons leads to increased levels of non-neuronal transcripts (Conaco et al., 2006), while increasing the expression of miR-124 in non-neuronal HeLa cells leads to a shift of the gene expression profile toward that of neuronal cells (Lim et al., 2005). A study by Makeyev et al. (2007) showed that miR-124 stimulated this shift by altering brain-specific alternative pre-mRNA splicing. During development of the mouse embryonic brain, miR-124 directly repressed the expression of polypyrimidine-tract-binding protein (PTBP), which represses global neuronal alternative pre-mRNA splicing in non-neuronal cells. During neuronal differentiation, miR-124 represses PTBP expression, initiating the transition from non-neuronal-specific to neuronal-specific alternative splicing transcript patterns. Interestingly, knock-down of PTBP expression can directly trans-differentiate mouse fibroblasts into functional neurons (Xue et al., 2013).

miR-124 has also been shown to be an important regulator of neuronal fate in the subventricular zone (SVZ). Inhibition of endogenous miR-124 maintained SVZ stem cells as dividing neural precursors, while overexpression of miR-124 dramatically increased production of neurons. Sox9 was proven to be a direct target gene of miR-124 relate to this function (Cheng et al., 2009). In addition, several other target genes have been identified as targets of miR-124 in relation to this function. For instance, in SVZ progenitor cells, miR-124 promotes stroke induced neurogenesis by repressing the JAG-Notch signaling pathway (Gu, 1980; Liu et al., 2011). miR-124 also stimulates neuronal differentiation by repressing ITGB1 and LAMC1, which are abundantly expressed in neural progenitor cells, but are down-regulated during neuronal differentiation in the chick embryos (Cao et al., 2007). In both chick and mouse embryos, SCP1 inhibits neurogenesis during developmental of the CNS, and miR-124 promotes neurogenesis in part by repressing of SCP1 (Visvanathan et al., 2007). In addition to its effects on determination of the fate of neurons, miR-124 is also implicated in the control of development of neurites during neuronal differentiation (Yu et al., 2008), and regulates process development by repressing RhoG expression (Franke et al., 2012).

In this study, we demonstrate that CBX2 is a negative regulator of neurite development. CBX2 transcpitionally inhibits the expression of several neuronal genes including miR-124. Inturn, miR-124 post-transcriptionally represses CBX2 expression and thus forming a negative feedback loop to regulate neuronal differentiation. It was found that PcG proteins epigenetically suppress the neuronal genes during neural stem cell neuronal differentiation (Hirabayashi et al., 2009), which indicates the important role of PcG proteins in the regulating of neuronal development, therefore our research reveals an important regulatory mechanism in this process.

## Materials and Methods

### Ethics Statement

This study was carried out in strict accordance with the recommendations in the Guide for the Care and Use of Laboratory Animals of the National Institutes of Health. The protocol was approved by the Committee on the Ethics of Animal Experiments of Southern Medical University (Permit Number: SYXK2011-0074).

### Cell and Culture Conditions

#### Cell Lines

BE(2)-M17(M17), SY5Y, and P19 cell lines were purchased from the American Type Culture Collection (Manassas, VA, United States). The M17 and SY5Y cells were maintained in Dulbecco’s modified Eagle’s medium (DMEM)/F12 (Gibco, Thermo Fisher Scientific, Inc., Waltham, MA, United States) supplemented with 10% fetal bovine serum (FBS; Gibco). The transfected M17 and SY5Y cells were differentiated with DMEM/F12 plus 1% FBS, 1% penicillin/streptomycin (Gibco), and 10 μM of retinoic acid (RA) at a density of 1 × 10^4^ cells/cm^2^ in poly-L-lysine-coated 24-well tissue culture dishes. The non-transfected M17 cells were differentiated in a similar way to the transfected M17 cells except that two additional small molecules, SB431542 (10 μM) and Db-cAMP (1 mM), were added to the culture medium to accelerate neuronal differentiation (Passeri et al., 2015). The P19 cells were maintained in α-modified minimum essential medium (α-MEM, Invitrogen, Carlsbad, CA, United States) supplemented with 10% FBS. Retinoic acid-induced P19 cell differentiation was performed using the procedures described previously with a slight modification (Lyu et al., 2003). Briefly, the P19 cells were first cultured in suspension at a seeding density of 1 × 10^5^ cells/ml in the presence of 1 μM of all-trans-RA in bacterial-grade Petri dishes to aggregate. After 4 days of aggregation, the cells were dissociated into single cells by trypsin-ethylenediaminetetraacetic acid and plated in a poly-L-lysine-coated tissue culture dish at a density of 1 × 10^5^ cells/cm^2^ in Neurobasal-A medium (Life Technologies, Darmstadt, Germany) supplemented with 2% B27 supplement (Life Technologies). The cells were allowed to attach for 24 h, after which 10 μM of cytosine arabinoside (Ara-C) was added to the culture medium for 24 h to inhibit proliferation of non-neuronal cells.

#### Primary Cortical Neurons

The primary cortical neurons were abstained from embryonic 16–17-day-old C57/BL mouse pups as described elsewhere (Lesuisse and Martin, 2002). The cells were cultured in poly-L-lysine-coated 24-well plates at a density of 250,000 cells/well in Neurobasal medium supplemented with 2% B27 (Life Technologies), 0.5 mM glutamine, and penicillin and streptomycin. Twenty-four hours after plating, the neurons were transfected with Lipofectamine 2000 reagent (Invitrogen) according to the manufacturer’s instructions. The cell morphology was examined 36 h after transfection, and the neurite length was determined using ImageJ software (NeuronJ, National Institutes of Health, Bethesda, MD, United States). A single neurite was manually selected and traced semi-automatically to measure neurite length. Neurons were deemed as bearing neurites if a process greater than one cell body diameters in length could be observed.

### Plasmid Construction

The hU6-hsa-miR-124-CMV-GFP plasmid for miR-124 overexpression was constructed by cloning the human primary miR-124-1 with approximately 50 bp flanking nucleotides and ligated with the BamHI and HindIII sites of hU6-MCS-CMV-GFP-SV40-Neomycin plasmid (Genechem, Shanghai, China) The primers were 5′-GTCTGGATCCTCC TTCCTCAGGAGAAAG-3′ (forward) and 5′-GTCTAAG CTTCCAAAAAACGCTGTTTGCATCTCTAAGC-3′ (reverse) The shRNA plasmid used in this study was constructed using the pGPU6/GFP/Neo plasmid (GenePharma, Shanghai, China), and the targeting sequences were GCTGG TCCTCCAAACATAACA (shCBX2-1) and GGCCTTCCAGAAGAAGGAACA (shCBX2-2) for the human CBX2 gene and TGTAGATGAAACCAAACCTAA (shGAP-43) for the human GAP-43 gene (Osswald et al., 2015) and AGATGAAGATAGTCAAGAA (shCBX4) for human CBX4 (Luis et al., 2011). The CBX2 3′ UTR reporter constructs were made by amplifying the human CBX2 3′ UTR sequence by polymerase chain reaction (PCR) and cloning into the XbaI site of the pGL3-control plasmid (Promega, Madison, WI, United States). The primers for the CBX2 3′-UTR were 5′-GTCTTCTAGAGTACCAGACTGCAAGACC-3′ (forward) and 5′-GTCTTCTAGAGTCTTCTAGAACGCTCTTGAAGCACCAG-3′ (reverse). Mutations of the miR-124 seed sites in the CBX2 3′ UTR were generated using the QuikChange site directed mutagenesis kit (Stratagene, La Jolla, CA, United States). The primers were 5′-GATCTTTTTAACAGTGTCGGTTT GGGGAGGGACCCATGTC-3′ (forward) and 5′-GACATGGGTCCCTCCCCAAACCGACACTGTTAAAAAGATC-3′ (reverse) for the first site and PCR and 5′-CTCCAGACCCTGATTCGGTCGGT TTCTGTTTACCAGCTAC-3′ (forward) and 5′-GTAGCTGGTAAACAGAA ACCGACCGAATCAGGGTCTGGAG-3′ (reverse) for the second site, with the sequences “GCC” in the seed sites of miR-124 mutated to “CGG” (underlined). For CBX2 and GAP-43 overexpression, the full-length coding region of CBX2 and full-length coding region with the 3′-UTR of the GAP-43 gene was cloned by PCR with cDNA amplified by reverse transcription-PCR using RNA isolated from M17 cells or the embryonic mouse brain and inserted into the pLVX-IRES-tdTomato vector (Clontech, Mountain View, CA, United States). The primers were 5′-GTCTCTCGAGTTCAACCTGAGGCATTACTG-3′ (forward) and 5′-GTCTGCGGCCGCACACGCTCTTGAAGCACCAG-3′ (reverse) for human CBX2 (using the XhoI and NotI restriction Enzyme cutting sites), 5′-GTCTGAATTCGCTTTGTGTGCAGCAGTGAGC-3′ (forward) and 5′-GTCTTCTAGAGGTCTGGCTCGGGCAATGGTC-3′ (reverse) for mouse CBX2 (using the RcoRI and XbaI restriction Enzyme cutting sites), and 5′-GTCTGAATTCGCGATGACAAAGTCCTGC-3′ (forward) and 5′-GTCTGGATCCACTC GATATTTTGGACTCC-3′ (reverse) for human GAP-43 (using the RcoRI and BamHI restriction Enzyme cutting sites), and 5′-GTCTGAATTCAGATACCACCATGCTGTG-3′ (forward) and 5′-GTCTGGATCCTTTGGACTCCTCAGAACG-3′ (reverse) for mouse GAP-43 (using the RcoRI and BamHI restriction Enzyme cutting sites).

### Generation of Adenoviruses and Infections

Adenovirus particles overexpressing miR-124 (VR207564) were obtained from Shandong Vigenebio, Co., Ltd. (Jinan, China). shRNA adenovirus particles for silencing CBX2 expression were obtained from GenePharma (Shanghai, China), the target sequence is similar to shCBX2-1.

M17 or SY5Y cells were plated in a six-well plate at 50% confluence. Before infection, the culture medium was removed from the plate wells and replaced with medium supplemented with Polybrene at a final concentration of 6 μg/ml. The cells were then infected by adding adenovirus particles (100 MOI) to the cell culture for 12 h, after which the medium was refreshed. Cells were collected 3 days later for ChIP assays (M17 cells) or for neuronal differentiation experiments (SY5Y cells).

### Luciferase Reporter Assay

M17 cells were placed in 48 wells and cotransfected with 0.05 μg of the firefly luciferase report vector, 0.4 μg of hU6-hsa-miR-124-CMV-GFP or hU6-hsa-miR-Scramble-CMV-GFP, and 0.01 μg of pMIR-REPORT β-gal control plasmid (Ambion, Austin, TX, United States) for normalization of transfection using the Lipofectamine 2000 reagent according to the manufacturer’s protocol. After 24 h, the luciferase activity was measured using the Bright-Glo luciferase assay system (Promega), and the β-galactosidase activity were measured using the high-sensitivity β-galactosidase assay kit (Agilent Technologies, Palo Alto, CA, United States). The luciferase activity was normalized against the β-Gal activity for the same cells. The experiments were performed in triplicate.

### Immunoblotting

The cells were washed with phosphate-buffered solution twice before being lysed using radioimmunoprecipitation assay buffer (0.15 M NaCl, 0.05 M Tris-HCl [pH 7.5], 1% Triton X-100, 0.1% sodium deoxycholate, 0.1% sodium dodecyl sulfate supplemented with protease inhibitor cocktail; Sigma-Aldrich, St. Louis, MO, United States). The protein concentrations of the cell extracts were measured using the bicinchoninic acid assay kit (Pierce, Rockford, IL, United States). Extracts of equal amounts were loaded and subjected to sodium dodecyl sulfate polyacrylamide gel electrophoresis followed by Western blot analysis. The following antibodies were used: CBX2 (1:2000, Abcam, Cambridge, MA, United States); CBX2 (1:2000, Zen-Bio, Inc., Research Triangle Park, NC, United States); GAP-43 (1:5000, Abcam); neuron-specific class III beta-tubulin (Tuj1; 1:5000, Beyotime Biotech, Jiangsu, China); H2AK119Ub (1:5000, Cell Signaling Technology, Beverly, MA, United States); a-Tubulin (1:2000, Sigma); and GAPDH (1:5000, Abcam).

### Immunocytochemistry

Differentiated SY5Y cells cultured in 24-well plates were fixed with 4% paraformaldehyde (PFA) for 15 min at room temperature, permeabilized with 0.3% TritonX-100 for 30 min, blocked with bovine serum albumin and incubated with the Tuj1 antibody at 4°C overnight. The cells were then incubated with the AlexaFluor 594 (Invitrogen) conjugated secondary antibody for about 1 h.

### Real-Time RT-PCR

Total RNA was isolated from M17 cells using the RNAiso reagent (TaKaRa, Dalian, China) according to the manufacturer’s instructions. For qRT-PCR of protein-coding genes, first-strand cDNA was reverse-transcribed from 500 ng of total RNA extract using ReverTra Ace qPCR RT Master Mix (Toyobo, Tokyo, Japan). SYBR Green-based qPCR (Toyobo) analysis was carried out using the Stratagene Mx3005P qPCR system (Agilent Technologies, Inc., Santa Clara, CA, United States). The abundance of transcripts of the housekeeping gene GAPDH was used as a loading control. The following primers were used: GAPDH, forward 5′-AGAAGGCTGGGGCTCATTTG-3′ and reverse 5′-AGGGGCCATCCACAGTCTTC-3′; CBX2, forward 5′-CAGCAAGAGGGAC TGTGTC-3′ and reverse 5′-GAGCTGCTACTCTCCTCAC-3′; GAP-43, forward 5′-AGGA TAAGGCTCATAAGG-3′ and reverse 5′-CATCATCCTTCTCCTTGG-3′; Ascl1, forward 5′-GCTGCC AAGCTGCTCAAC-3′ and reverse 5′-TCGTCGTCTTCGTCAGCA-3′; Ngn1, forward 5′-GCTCTCTGACCCCAGTAGC-3′ and reverse 5′-GCGTTGTGTGGAGCAAGTC-3′; Ngn2, forward 5′-CCAACTAAGATGTTCGTC-3′ and reverse 5′-CTCCTCCTCTTCTTCGTC-3′; NeuroD1, forward 5′-CCTTCCTTTGATGGACCCC-3′ and reverse 5′-GATTGATCCGT GGCTTTGG-3′; and NeuroD2, forward 5′-GACTACAACAGCTCCGAG-3′ and reverse 5′-GTAGTG CATAGAGTAGTG-3′. The miRNA levels were measured by qRT-PCR using a miDETECT A Track^TM^ miRNA qRT-PCR kit (RiboBio, Guangzhou, China). The primers for miR-124, miR-9, and U6 small nuclear RNA were obtained from RiboBio. The miRNA expression levels were normalized to the expression of the internal control (U6) using the 2^-ΔΔC_T_^ method.

### Chromatin Immunoprecipitation (ChIP)

M17 cells were first cross-linked in fresh culture medium containing 1% formaldehyde for 10 min at room temperature; 0.1 ml of 1.25 M glycine was then added to stop fixation and the cells lysed in lysis buffers. Chromatin was sonicated to an average size of 0.5–2 kb. We used an ultrasonic processor (UP200S, Hielscher Ultrasonics GmbH, Teltow, Germany) and sonicated at 30% amplitude for 20 × 1-s pulses (with a 0.5-s pause between pulses) while the samples were in an ice bath. The processed whole-cell extract was incubated overnight at 4°C with 100 μl of Protein G beads that had been preincubated with 2 μg of the appropriate antibodies. Ten percent of the chromatin used for each chromatin immunoprecipitation reaction was kept as input DNA. The beads were washed three times with radioimmunoprecipitation assay buffer and once with TE containing 50 mM NaCl. Bound complexes were eluted from the beads by heating at 65°C with frequent vortexing, and crosslinking was reversed by overnight incubation at 65°C. Input whole-cell extract DNA (reserved from the sonication step) was also treated for crosslink reversal. The immunoprecipitated DNA and whole-cell extract DNA were then purified by treatment with RNaseA, proteinase K, and a phenol:chloroform:isoamyl alcohol extraction. Purified DNA samples were normalized and subjected to qPCR analysis. The antibodies used for pull-downs were anti-CBX2 (Santa Cruz Biotechnology, Dallas, TX, United States) and anti-H2AK119Ub (Cell Signaling Technology). Quantitative RT-PCR was performed using a SYBR Premix Ex Taq GC kit (Perfect Real Time, TaKaRa). The following primers were used for qPCR: Ascl1, forward 5′-GCACTGCAACAACAAACC-3′ and reverse 5′-CCTTTTCAATGGGACACC-3′; GAP-43, forward 5′-GACTGACTACAATTCCTC-3′ and reverse 5′-GA TATTCCAGTCAATCC-3′; miR-9, forward 5′-GGAATGCCCTAAGCCTTC-3′ and reverse 5′-CGCTTTGGAACTTG ATAG-3′; miR-124, forward 5′-TCATTGTCTGGAGCTGCA-3′ and reverse 5′-CATCCTTTCGCATCCAGG-3′; NeuroD1, forward 5′-TCTAACTGGCGACAGATG-3′ and reverse 5′-CAGCCAAGAGCCTGGAAC-3′; NeuroD2, forward 5′-TCGCTTTCCCTGCGTGTG-3′ and reverse 5′-AGCTATCACATGAGAGAC-3′; Ngn1, forward 5′-CATAAATTATGCAAATAC-3′ and reverse 5′-GAGTGTGGCACACGACTG-3′; Ngn2, forward 5′-ACCATTTTATGTTGATTAG-3′ and reverse 5′-CATTATCATCATTCATTTC-3′; and Gata1, forward 5′-ACATACACAGGAGTCTAG-3′ and reverse 5′-TGTAGTGTCCAGACAAGC-3′.

### Statistical Analysis

All of the experiments were repeated at least three times. Neurite lengths were determined using ImageJ software (NIH). Single neurites was manually selected and traced semi-automatically to measure neurite length. The identified neurons were scored as having neurites if a process greater than one cell body diameters in length could be seen. The results were analyzed using one-way analysis of variance combined with multiple comparison or the unpaired *t*-test to detect significant differences between the experimental groups. *P* < 0.05 was considered statistically significant.

## Results

### CBX2 Inhibits Neuronal Differentiation in Various Neuronal Differentiation Models

**FIGURE 1:**
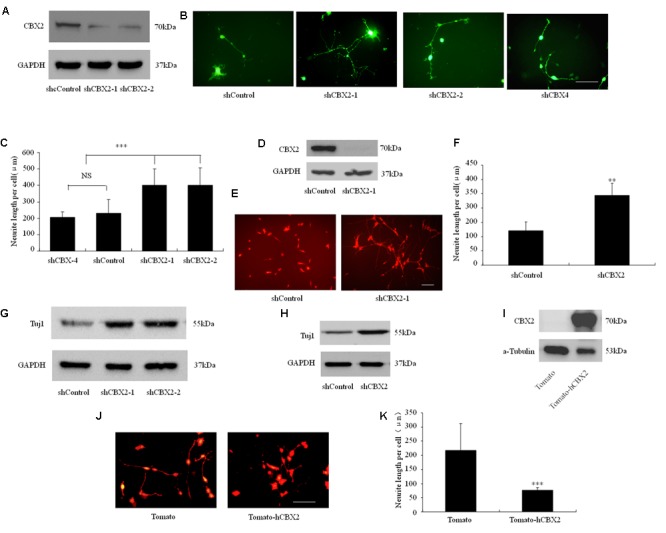
CBX2 is a negative regulator of neuronal differentiation. **(A)** M17 cells were co-transfected with each construct as indicated in the image and the MCS-EF1-copGFP-T2A-puro vector (System Biosciences) by using Lipofectamine 2000 reagent. Puromycin was added to the culture medium 24 h later at a concentration of 1 μg/ml to eliminate non-transfected cells. Immunoblotting analysis was performed 72 h later. Immunoblotting for CBX2 expression showed that shCBX2-1 and shCBX2-2 reduced the amount of endogenous CBX2 in M17 cells. **(B)** The morphology of the transfected M17 cells was monitored on the 3rd day after differentiation by observing the GFP fluorescence. **(C)** Knocked-down of CBX2 increased the neurite length in differentiated M17 cells. Scale bar: 100 μm. The data are presented as mean ± SD, *n* = 30 cells, ^∗∗∗^*P* < 0.001. **(D)** Immunoblotting for CBX2 expression showed that adenoviruses expressing shCBX2-1reduced the amount of endogenous CBX2 in SY5Y cells. **(E)** SY5Y cells infected with shRNA adenoviruses particles were labeled with anti-Tuj1 antibody and stained with an Alexa 594-conjugated secondary antibody. **(F)** CBX2 knocked-down increased neurite length of differentiated SY5Y cells. Scale bar: 100 μm. The data are presented as the mean ± SD, *n* = 30 cells, ^∗∗^*P* < 0.01. **(G)** Immunoblotting for Tuj1 expression showed that shCBX2-1 and shCBX2-2 increased the amount of endogenous Tuj1 expression in M17 cells. **(H)** Immunoblotting for Tuj1 expression showed that shCBX-1 increased the amount of endogenous Tuj1 expression in SY5Y cells. **(I)** Validation of CBX2 protein expression of the indicated constructs detected by Western Blotting using the anti-CBX2 antibody. **(J)** The morphology of the transfected M17 cells was monitored on the 3rd day after differentiation by observing the RFP (Tdtomato) fluorescence. **(K)** Overexpression of CBX2 decreased the neurite length in differentiated M17 cells. The data are presented as mean ± SD, *n* = 30 cells, ^∗∗∗^*P* < 0.001.

PcG proteins are known to epigenetically suppress the Ngn1 locus during neuronal differentiation of neural stem cells, especially in the astrogenic phase, and regulate the duration of the neurogenic phase in the developing neocortex (Hirabayashi et al., 2009). However, although the important role of PRC1 in regulating neuronal development is understood, how its components and functions are regulated during neuronal differentiation is still unclear. Recently, NRSF/REST was found to recruit PRC1 to the promoter region of some neuronal genes and to show a strong interaction with the PRC1 component CBX2 (Ren and Kerppola, 2011). Therefore, we embarked on a study of the role of the CBX2 gene in neuronal differentiation. We performed loss-of-function and gain-of-function experiments in differentiated neuronal linage cells. For the loss-of-function experiments, we used the independent shRNA constructs shCBX2-1 and shCBX2-2 to reduce the amount of CBX2 endogenously expressed in M17 cells. As shown in **Figure 1A**, overexpression of shCBX2-1 or shCBX2-2 greatly reduced the endogenous expression of CBX2 in M17 cells. To determine the role of the CBX2 gene in neuronal differentiation, we first transfected human neuroblastoma BE(2)-M17 lines with the shRNAs to investigate their effect on neuronal differentiation. Knock-down of CBX2 with shCBX2-1 or shCBX2-2 but not a previously validated CBX4 shRNA significantly stimulated neurite outgrowth in differentiated M17 cells (**Figures 1B,C**). In addition, we used adenovirus to mediate the expression of shCBX2-1 to reduce CBX2 expression in SY5Y cells (**Figure 1D**) and found that knock-down of CBX2 could also promote the neurite development of SY5Y cells (**Figures 1E,F**). We also examined the Tuj1 level in these cells. As show in **Figures 1G,H**, knock-down of CBX2 lead to increased expression of Tuj1, which suggests a more differentiated state. For the gain-of-function experiments, we introduced the CBX2 overexpression constructs into differentiated M17 cells. Overexpression of CBX2 greatly inhibited neurite outgrowth in M17 cells (**Figures 1I–K**), suggesting CBX2 is a negative regulator of neurite development.

### CBX2 Is Down-Regulated During Neuronal Differentiation

**FIGURE 2:**
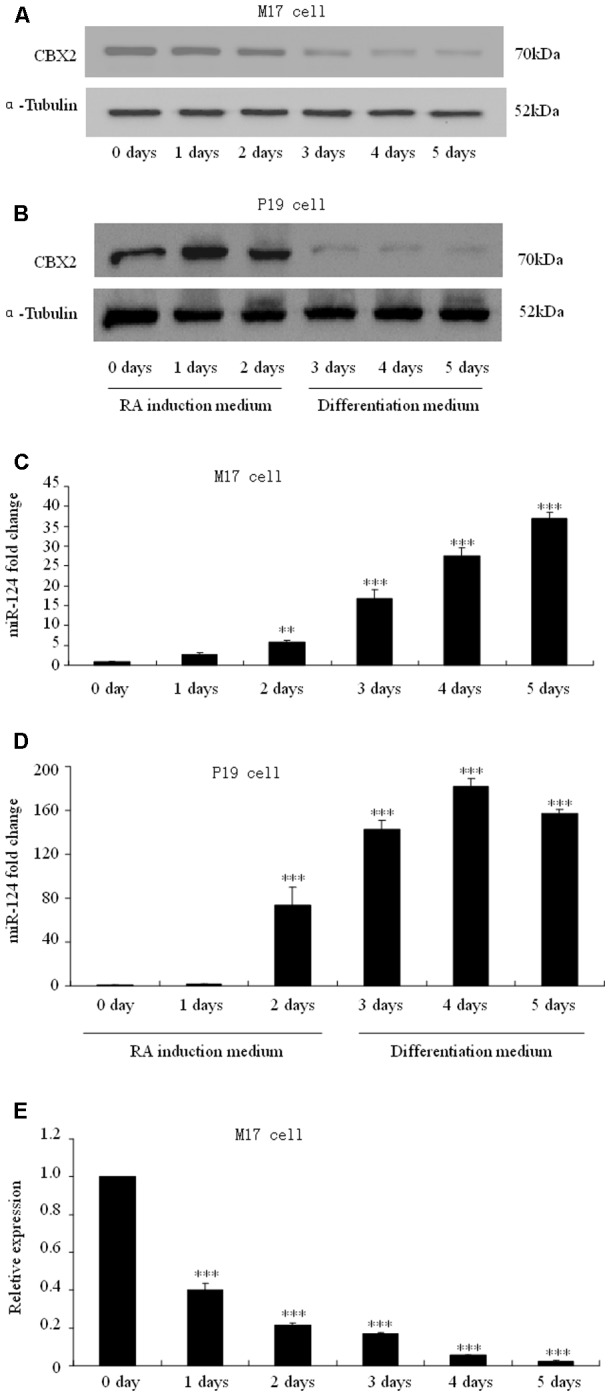
CBX2 and miR-124 expressions are inversely correlated during neuronal differentiation. The immunoblotting analysis is shown of CBX2 expression during human M17 cell **(A)** and mouse P19 cell **(B)** neuronal differentiation. Real-time polymerase chain reaction (PCR) analysis of miR-124 expression during neuronal differentiation of human M17 cells **(C)** and mouse P19 cells **(D)** is shown. **(E)** RT-qPCR analysis of CBX2 mRNA expression during M17 cells neuronal differentiation. The data are presented as the mean ± SD, *n* = 3 independent experiments, ^∗∗^*P* < 0.01, ^∗∗∗^*P* < 0.001.

To determine how CBX2 was regulated during neuronal differentiation, we took advantage of RA-induced human M17 cells and mouse P19 embryonic carcinoma cells as *in vitro* differentiation model. We found that the CBX2 protein level was noticeably down-regulated in M17 cells (**Figure 2A**) and P19 cells (**Figure 2B**) undergoing neuronal differentiation. Given that microRNAs has recently been reported to have an important role in regulating cell development, we next predicted the potential miRNA–mRNA interaction using online TargetScan software^1^ and found that the 3′-UTR of the CBX2 gene comprises two miR-124-binding sites. Indeed, we found that expression of CBX2 protein was inversely correlated with expression of miR-124 (**Figures 2C,D**). These observations suggest the possibility of a direct interaction between CBX2 and miR-124 in neuronal differentiation. We also examined the mRNA changes of CBX2 during M17 cell neuronal differentiation and found a decrease in CBX2 mRNA in this process (**Figure 2E**) which means miR-124 may regulate CBX2 expression by inducing CBX2 mRNA degradation.

### CBX2 Is a Direct Target Gene of miR-124

**FIGURE 3:**
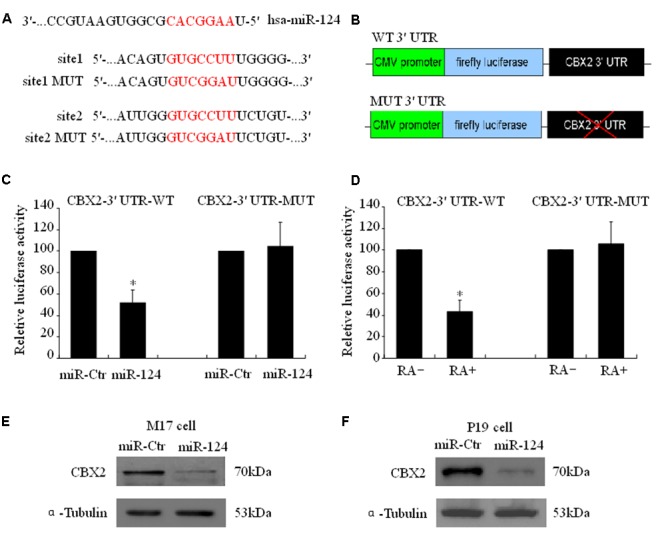
CBX2 mRNA is a direct target of miR-124. **(A)** Predicted configurations of duplex formations between miR-124 and its target sites in the 3′ UTR of CBX2. **(B)** Schematic representation of human CBX2 3′ UTR, with wild-type (WT) or mutated (MUT) miR-124 binding sites, subcloned to the downstream of PGL3 firefly luciferase reporter plasmid. **(C)** Quantification of reporter activity after transfection of the indicated constructs in M17 cells. The data are presented as mean ± SD, *n* = 3 independent experiments, ^∗^*P* < 0.05. **(D)** Quantification of reporter activity 5 days after transfection of the indicated constructs in RA treated or untreated M17 cells. The data are presented as mean ± SD, *n* = 3 independent experiments, ^∗^*P* < 0.05. Immunoblot shows that miR-124 induces a decrease in CBX2 protein levels in both M17 cells **(E)** and P19 cells **(F)**.

Using a firefly luciferase reporter assay, we then tested whether the predicted miR-124 binding sites in the CBX2 3′-UTR (**Figure 3A**) were sufficient for miR-124-mediated inhibition of expression. The 3′-UTR of CBX2 was inserted into the XbaI site of a pGL3-control plasmid (CBX2-3′-UTR, **Figure 3B**). Cotransfection of CBX2-3′-UTR with a miR-124 expression plasmid (hU6-hsa-miR-124-CMV-GFP, miR-124) significantly reduced the luciferase activity when compared with co-transfection with the control expression plasmid (hU6-scrambled-CMV-GFP, miR-ctr). When the miR-124 binding sites in the CBX2-3′-UTR was mutated (“GUGCCUU,” the underlined nucleotides were mutated to “CGG,” **Figure 3A**), the inhibitory effect of miR-124 on CBX2-3′-UTR luciferase activity disappeared (**Figure 3C**). In addition, we found that compared with the undifferentiated group, RA induced M17 cell neuronal differentiation could significantly reduce the luciferase activity of the wild-type (WT) CBX2-3′-UTR construct but has no effect on the mutated (MUT) CBX2-3′-UTR construct (**Figure 3D**). To further confirm CBX2 as a direct cellular target of miR-124, we transfected human M17 cells and mouse P19 cells with the miR-124 expression plasmid hU6-hsa-miR-124-CMV-GFP or the negative control plasmid hU6-scrambled-CMV-GFP; these were then further selected by cell sorting based on GFP expression using flow cytometry 3 days after transfection. As shown in **Figures 3E,F**, overexpression of miR-124 dramatically reduced the endogenous CBX2 protein levels when compared with the negative control plasmid. These results indicate that CBX2 may be a direct target of miR-124.

### CBX2 Inhibits the Neurite Stimulating Effect of miR-124

**FIGURE 4:**
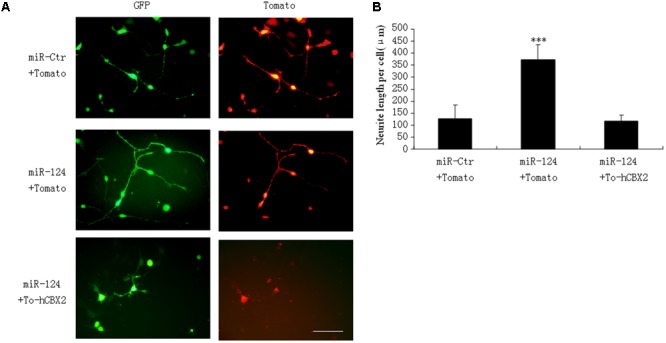
CBX2 over-expression inhibits the neurite stimulating effect of miR-124. **(A)** The morphology of M17 cells transfected with the indicated vectors was monitored by Td-tomato fluorescence. **(B)** Quantification of the neurite length of the indicated groups is shown. Scale bar: 100 μm. The data are presented as mean ± SD, *n* = 30 cells, ^∗∗∗^*P* < 0.001.

Our research and that of other groups has identified that miR-124 stimulates neuronal differentiation (Makeyev et al., 2007; Gu et al., 2014). To address the functional significance of miR-124-induced downregulation of CBX2 during neuronal differentiation, M17 cells were co-transfected with the indicated overexpressing plasmids before neuronal differentiation (**Figure 4A**). The neurites on M17 cells with miR-124 overexpression were longer than those on control cells 1 day after neuronal differentiation, and simultaneous overexpression of CBX2 significantly inhibited the stimulating effect of miR-124 on neurite outgrowth (**Figures 4A,B**), meaning that CBX2 is an important target gene in miR-124-mediated regulation of neuronal development.

### CBX2 Binds Directly to the Promoter Region of Several Neuronal Genes

**FIGURE 5:**
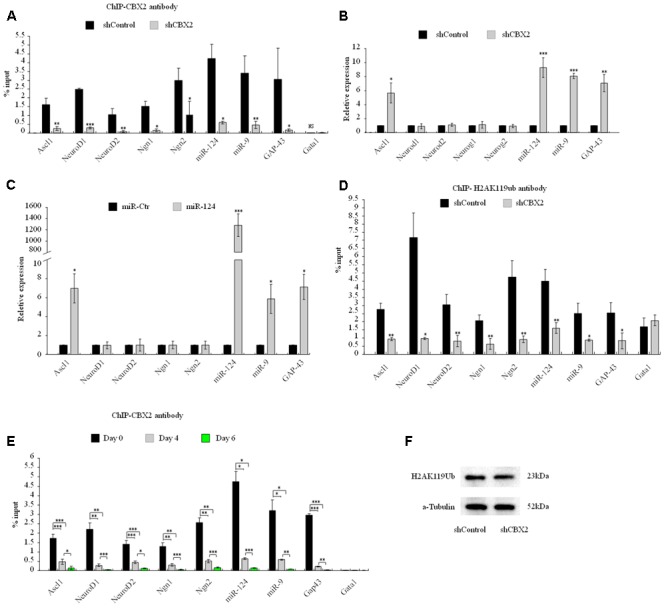
CBX2 directly binds to the promoter of several neuronal genes and regulates the expression of some of them. **(A)** ChIP-qPCR analysis of the enrichment of CBX2 at selected gene promoters in control and CBX2 knocked-down M17 cells. The data are presented as mean ± SD, *n* = 3, ^∗∗∗^*P* < 0.001. **(B)** RT-qPCR analysis of selected genes in control and CBX2 knocked-down M17 cells. The data are presented as mean ± SD, *n* = 30, ^∗^*P* < 0.05, ^∗∗^*P* < 0.01, ^∗∗∗^*P* < 0.001. **(C)** RT-qPCR analysis of selected genes in control and miR-124 over-expressed M17 cells. The data are presented as mean ± SD, *n* = 3, ^∗^*P* < 0.05, ^∗∗∗^*P* < 0.001. **(D)** ChIP-qPCR analysis of the enrichment of H2AK119Ub at selected gene promoters in control and CBX2 knocked-down M17 cells. The data are presented as mean ± SD, *n* = 3, ^∗∗^*P* < 0.01, ^∗∗∗^*P* < 0.001. **(E)** ChIP-qPCR analysis of the enrichment of CBX2 at selected gene promoters in during M17 cells neuronal differentiation. The data are presented as mean ± SD, *n* = 3, ^∗^*P* < 0.05, ^∗∗^*P* < 0.01, ^∗∗∗^*P* < 0.001. **(F)** Immunoblotting analysis is shown of H2AK119Ub expression in control and CBX2 knocked-down M17 cells.

Next we sought to investigate the molecular mechanism underlying the CBX2-mediated neuronal developmental effect. ChIP-Sequencing (ChIP-Seq) dates from Morey et al. (2012) showed that CBX2 binds to the regulatory region of many genes on the chromosome when embryonic stem cells differentiated into embryoid bodies. Among these genes, we identified many neuronal genes, including Ascl1, NeuroD1, NeuroD2, Ngn1, Ngn2, GAP-43, miR-124, and miR-9. CBX2 is one of the most abundant genes expressed in neural stem cells (Hou et al., 2017), so it is reasonable to speculate that CBX2 may regulate neuronal gene expression during neuronal differentiation. By knocking down the expression of CBX2 in M17 cells, we verified by ChIP-qPCR that CBX2 is present at the promoter region of these neuronal genes but not at the negative control promoter region of Gata1 gene (Lau et al., 2017) (**Figure 5A**). We also examined the expression of these genes using q-PCR and found that four of them (Ascl1, miR-124, miR-9, and GAP-43) were upregulated after CBX2 was knocked-down (**Figure 5B**) or miR-124 was overexpressed (**Figure 5C**); the expression of the remaining four neuron-specific genes were unchanged, suggesting that their expression may require additional CNS-specific activators that are not expressed after CBX2 is knocked-down under our differentiation conditions. In addition, the interactions of CBX2 with these promoters were decreased during M17 cell neuronal differentiation (**Figure 5D**) which was consistent with the downregulation of CBX2 during neuronal differentiation (**Figures 2A,B**). Given that CBX family protein-mediated recruitment of PRC1 complexes can catalyze ubiquitylation of histone H2A on lysine 119 (H2AK119Ub), we next examined the H2AK119Ub modifications around the promoter region of these genes using ChIP-qPCR and found H2AK119Ub was also decreased at these promoters after CBX2 was knocked-down (**Figure 5E**). We further examined the global H2AK119Ub level but found no differences between the control and CBX2 knock-down groups (**Figure 5F**), suggesting that changes in H2AK119Ub resulting from reduction of CBX2 may be specific to particular genes.

### CBX2 Regulates Neurite Development by Repressing GAP-43 Expression

**FIGURE 6:**
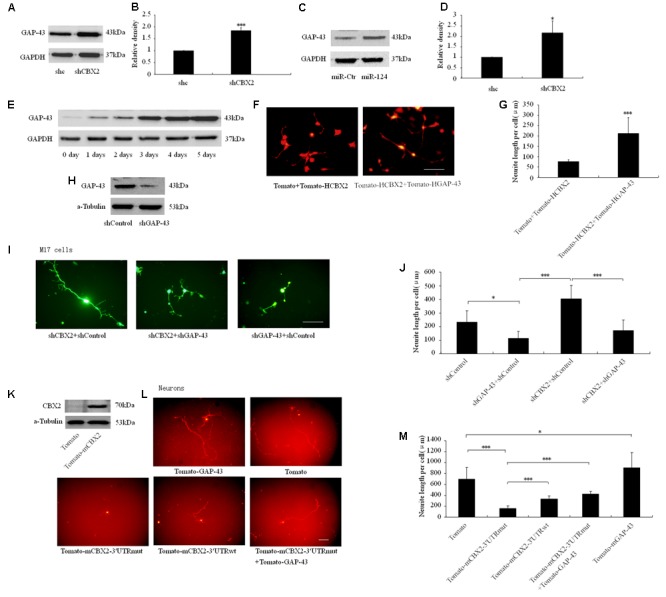
CBX2 regulated neurite development involves GAP43. Immunoblotting analysis **(A)** and quantification result **(B)** shown that CBX2 knocked-down induces an increase in GAP-43 protein levels. Immunoblotting analysis **(C)** and quantification result **(D)** shown that miR-124 overexpression induces an increase in GAP-43 protein levels. **(E)** An immunoblot shows that an increase in GAP-43 protein levels during M17 cell neuronal differentiation. **(F)** Cell morphology of M17 cells transfected with the indicated constructs was monitored on the 3rd day after neuronal differentiation by observing the Td-tomato fluorescence. Scale bar: 100 μm. **(G)** GAP-43 overexpression rescued the neurite inhibting effect of CBX2 overexpression in differentiated M17 cells. The data are presented as mean ± SD, *n* = 30 cells, ^∗∗∗^*P* < 0.001. **(H)** Immunoblotting for GAP-43 expression showed that shGAP-43 reduced the amount of endogenous GAP-43 in M17 cells. M17 cells were co-transfected with each construct as indicated in the picture and the MCS-EF1-copGFP-T2A-puro vector by using Lipofectamine 2000 reagent. Puromycin was added to the culture medium 24 h later at a concentration of 1 μg/ml to eliminate non-transfected cells. Immunoblotting analysis was preformed 72 h later. **(I)** Cell morphology of M17 cells transfected with the indicated constructs was monitored on the 3rd day after neuronal differentiation by observing the GFP fluorescence. Scale bar: 100 μm. **(J)** GAP-43 knocked-down attenuated the neurite stimulating effect of CBX2 silencing in differentiated M17 cells. The data are presented as mean ± SD, *n* = 30 cells, ^∗∗∗^*P* < 0.001. **(K)** Validation of mouse CBX2 protein expression of indicated constructs detected by Western Blot using the anti-CBX2 antibody in P19 cells. **(L)** The morphology of primary neurons transfected with the indicated vectors was monitored by Td-tomato fluorescence. **(M)** Quantification of the neurite length of the indicated groups is shown. Scale bar: 100μm. The data are presented as mean ± SD, *n* = 30 cells, ^∗^*P* < 0.05, ^∗∗∗^*P* < 0.001.

Overexpression of GAP-43 is generally associated with increased neurite growth in neurons (Donnelly et al., 2013; Williams et al., 2016), so we focused on this gene as the downstream effector of CBX2-regulated neuronal differentiation. The protein level of GAP-43 was increased after CBX2 was knocked-down and miR-124 was overexpressed in M17 cells (**Figures 6A–D**) and during the process of M17 cell neuronal differentiation (**Figure 6E**). In M17 cells, overexpression of GAP-43 rescued the neurite inhibiting effect of CBX2 overexpression (**Figures 6F,G**), while knockdown of GAP-43 using a previously validated shRNA sequence (Osswald et al., 2015) greatly reduced the endogenous expression of GAP-43 (**Figure 6H**) and repressed neurite elongation and attenuated the neurite-promoting effect of CBX2 silencing in differentiated M17 cells (**Figures 1B, 6I,J**). In embryonic mouse cortical neurons which were closer to the normal physiological state, overexpression of GAP-43 significantly promoted neurite elongation. CBX2 with 3′-UTR containing the mutated miR-124 binding sites significantly repressed neurite elongation compared with control and CBX2 with its wild-type 3′-UTR, while this could be partially rescued by combined with overexpression of GAP-43 (**Figures 6K–M**). These data demonstrate that CBX2 reduces neurite elongation at least partially via regulating expression of GAP-43.

## Discussion

**FIGURE 7:**
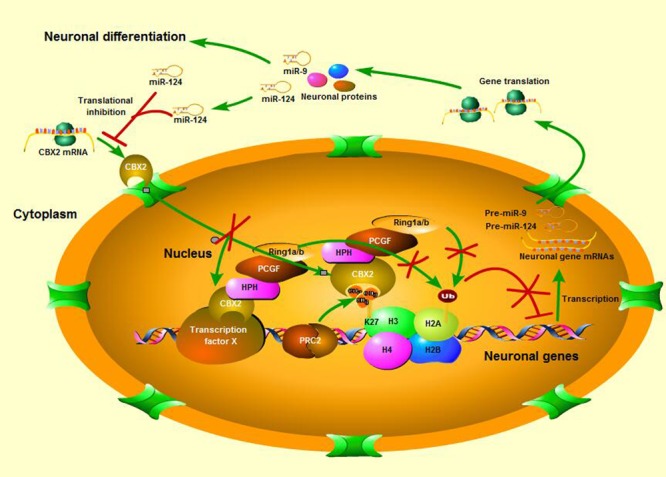
A schematic is shown of neuronal differentiation mediated by the miR-124 regulated CBX2 pathway.

In this study we identified CBX2 to be a novel regulator in neuronal differentiation and a direct target for miR-124-dependent gene expression regulation (**Figure 7**). We also discovered a precise molecular pathway for this regulation, i.e., miR-124 suppressed CBX2 expression to stimulate neurite development by upregulating expression of GAP-43. These findings contribute to the growing literature on the role of the PcG proteins in regulating neuronal development. The PRC1 PcG complex was reported to restrict neurogenic competence of neural progenitor cells, and inactivation of PcG by knockout of the Ring1B gene prolonged the neurogenic phase of neural progenitor cells and delayed the onset of the astrogenic phase (Hirabayashi et al., 2009). There are at least six distinct groups of mammalian PRC1 complexes, i.e., PRC1.1–1.6, each comprising one of six PcG RING fingers (Gao et al., 2012), and the E3 ligase RING1A/B. Further diversification arises from the mutually exclusive association of RING1A/B with either RYBP/YAF2 or one of the CBX proteins (Gao et al., 2012; Tavares et al., 2012; Morey et al., 2013), which bind H3K27me3 through their chromodomains. Ring1B is necessary for almost all the PRC1 subgroups to perform their function (Sauvageau and Sauvageau, 2010), but which PRC1 subgroup actually plays the neuron-regulating role and how its components are regulated during neuronal differentiation is unclear. In our present study, we found that CBX2, a number of CBX family proteins, was down-regulated during neuronal differentiation and knock-down of its expression promoted neuronal development, while its overexpression has the opposite effect. A recent study revealed CBX2 to be one of the most abundantly expressed genes in neural progenitor cells and its absence caused a significant reduction in generation of PAX6-positive neural progenitor cells from human embryonic stem cells (Xue et al., 2013). PAX6 are expressed by radial glial cells that have the intrinsic fate determinant of the neurogenic potential (Gotz et al., 1998; Englund et al., 2005). Interestingly, another study found PAX6 to be an upstream inhibitor of miR-124 expression (Fang et al., 2014). All these observations suggest that CBX2 and its regulated signal pathways may be required for generation and maintenance of neuronal progenitor cells and prevent their differentiation by forming large and complicated negative feedback mechanisms with miR-124 during development of the CNS.

miR-124 is an evolutionary conserved miRNA expressed in differentiating and mature neurons (Lim et al., 2005) and has been implicated in the regulation of neuronal gene expression; however, its cellular role is not completely understood. We and others have already identified a role for miR-124 in neurite development. Makeyev et al. (2007) reported that miR-124 promoted neurite outgrowth in neuroblastoma cells and that it stimulated neuronal differentiation in RA-treated and aggregated P19 cells. They found that the mRNA for the splicing factor PTBP1 is a direct target of miR-124 repression, and that part of the effects of miR-124 could be attributed to alternative mRNA splicing arising from downregulation of PTBP1; our previous study demonstrated that miR-124 repressed ROCK1 expression and stimulated neurite development via the PI3K/AKT signal pathway (Gu et al., 2014). In this study, the epigenetic regulator CBX2 was found to be another important target of miR-124 in the regulation of neurite development, suggesting miR-124 regulates neurite development by influencing multiple pathways. It is probably that many relevant targets remain to be identified, and their signaling hierarchies will need to be investigated extensively to understand how multiple signals are integrated in one single coordinated biological process.

Expression of miR-124 is thought to be regulated by REST (Conaco et al., 2006). This zinc-finger repressor negatively regulates many neuronal genes in stem cells, progenitors, and non-neuronal cells (Ballas et al., 2005). Potential REST binding sites are located near all three miR-124 loci (Conaco et al., 2006), and upregulation of miR-124 is probably a result of de-repression of the REST repressor complex during neuronal differentiation. Indeed, miR-124 expression is upregulated in Rest^+/-^ embryonic stem cell lines (Singh et al., 2008). Recent research found that PRC1 can be recruited to REST binding sites independently of CpG islands (Dietrich et al., 2012) and the H3K27Me3 mark, and CBX2 has been confirmed to bind directly to REST (Ren and Kerppola, 2011). In our study, we also found an interaction between CBX2 and the gene regulatory region of miR-124. Thus, it is likely that by targeting CBX2, miR-124 may reinforce its own expression in neuronal differentiation by repressing the inhibitory activity of REST, and this needs further investigation.

In this study, we found that CBX2 interacts with the promoter region of several neuro-associated genes, including Ascl1, NeuroD1, NeuroD2, Ngn1, Ngn2, miR-124, miR-9, and GAP-43. However, by knocking down the expression of CBX2 or overexpression of miR-124, we only detected changes in the expression of four of these genes, i.e., Ascl1, miR-124, miR-9, and GAP-43. The change in Ascl1 expression is consistent with the finding by Neo et al. (2014) that Ascl1 is one of the genes consistently de-repressed by miR-124 in N2a neuroblastoma cells. Nevertheless, Visvanathan et al. (2007) reported that overexpression of miR-124 induces expression of Ngn2 and NeuroD1 in P19 cells. These discrepancies could be attributable to differences in the type of cell culture conditions used or even to the different differentiation stages examined. Indeed, in the CNS, *in situ* hybridization to serial sections and double-labeling experiments indicate that different proneural genes are expressed in adjacent and non-overlapping regions of the neuroepithelium that correspond to future functionally distinct areas of the brain (Ma et al., 1997). Therefore, it is reasonable to speculate that downregulation of CBX2 and relief of its binding to the proneural gene’s promoter region only gives these genes a transcriptional permissive chromosomal state, and their expression may require additional CNS-specific activators that are expressed in different neuronal differentiation situations or stages. In addition, PRC1 can also be recruited to chromatin in a PRC2-independent manner (Sauvageau and Sauvageau, 2010; Yu et al., 2012). In these cases, different transcription factors facilitate localization of PRC1 to chromatin and regulate gene expression in different cellular context. Whether this is the case with CBX2 in neuronal differentiation remains to be determined.

Precise spatial and temporal control of GAP-43 protein levels is achieved via multiple mechanisms and is critical for GAP-43 function. The GAP-43 gene is transcribed in response to specific transcription factors (Chiaramello et al., 1996; Diolaiti et al., 2007; Tedeschi et al., 2009). All these transcription factors facilitate GAP-43 expression during neuronal differentiation, but how this gene is silenced before neuronal differentiation is not well-understood. In this study, we showed that CBX2 negatively regulates expression of the GAP-43 gene: knocking down the expression of CBX2 reduced the H2AK119Ub level around the GAP-43 gene promoter region and promoted GAP-43 gene expression. H2AK119Ub is thought to contribute to transcriptional repression by restraining elongation of RNA pol II (Simon and Kingston, 2009) and its downregulation seemed to account for the increased gene expression in our study. However, a recent study demonstrated that CBX2 represses gene expression as a result of its ability to compact the nucleosome but not to assemble the PRC1 protein complex (Lau et al., 2017). Therefore, the mechanism underlying gene upregulation with CBX2 knocked-down in neuronal development remains elusive and needs further investigation. In addition, the stability of GAP-43 mRNA is increased by HuD, a neuronal ELAV family mRNA-binding protein, binding the 3′ UTR of GAP-43 mRNA (Chung et al., 1997; Mobarak et al., 2000)and decreased by KSRP, a KH-type splicing regulatory protein, competing with HuD for binding (Bird et al., 2013). Further, localization of GAP-43 mRNA to axons in the dorsal root ganglia is regulated by the mRNA-binding protein IMP1/ZBP1 (Donnelly et al., 2011). Therefore, our findings indicate a novel transcriptional mechanism for regulating GAP-43 expression that may contribute to the precise control of GAP-43 expression during neuronal development.

In summary, we have provided evidence that CBX2 negatively regulates neuronal development. miR-124, through direct repression of CBX2, ensures the de-repression of neuronal genes and intrinsically regulates neuronal development. miR-124 is important in maintaining SVZ homeostasis because it regulates the number of progenitors and the timing of neuronal differentiation (Cheng et al., 2009). It has recently been suggested that brain tumor stem cells may be derived from neural stem cells (Sanai et al., 2005) and methylation levels of CpG loci near CBX2 were found to be higher in the tumor tissues of long-term survivors than in those of short-term survivors of glioblastoma (Zhang et al., 2013), suggesting that CBX2 may be upregulated and potentially have a stimulating effect on the progression of glioma. Thus, understanding the neurogenic effects mediated by miR-124 and its target genes may lead to novel therapeutic strategies for both neurodegenerative diseases and intractable brain tumors.

## Author Contributions

XJ, XG, XW, and XZ designed the content of the research project. XG, DS, XS, LL, QW, SLiu, PZ, and SLi finished all the experiments. XG and XJ wrote the article.

## Conflict of Interest Statement

The authors declare that the research was conducted in the absence of any commercial or financial relationships that could be construed as a potential conflict of interest.

## Footnotes

1 http://www.targetscan.org/
